# The Book World of Medicine and Science

**Published:** 1905-02-25

**Authors:** 


### THE BOOK WORLD OF MEDICINE AND SCIENCE.

WALSHAM'S HANDBOOK OF SURGICAL PATHOLOGY. Third Edition. By HERBERT J. PATERSON, M.B, B.C. Cantab., F.R.C.S.Eng. (London: Baillière, Tindall and Cox. 1904. Pp. 529. Price 10s. 6d. net.)

MR. PATERSON has done a good service, especially to Bart.'s men, in preparing a new edition of this excellent pathological work. The two previous editions were intended for use in the Museum of St. Bartholomew's Hospital, but in the present instance the scope has been widened, and to illustrate the descriptions given, specimens in the Royal College of Surgeons' Museum are referred to as well as those in the museum at Bart's. As might be expected, seeing that the previous edition was published so long ago as 1889, a great portion of the work has been rewritten. Although primarily intended for use in a pathological museum where ready reference may be made to actual specimens, the work affords an excellent arm-chair text-book of surgical pathology. Nevertheless, it is to be hoped that wherever possible the only correct method of learning pathology will be followed, that is to say by reading the book in the presence of morbid specimens that may be referred to for purposes of verifying the author's description.

THE SURGICAL TREATMENT OF FACIAL NEURALGIA. By J. HUTCHINSON, JUN., F.R C.S. (London: John Bale, Sons, and Danielsson, Limited. 1905. Pp. 151. Price 7s. 6d. net).

THIS book is almost entirely devoted to the subject of epileptiform neuralgia. Though fortunately rare, there is no kind of case which will plague the medical practitioner more than trigeminal neuralgia. As Mr. Hutchinson points out, the only proper treatment for this complaint, once it has become established, is by operation. In the earlier stages, when the pain is confined to the distribution of one of the main branches of the fifth nerve an extracranial neurectomy may be performed, on the ground that temporary relief will be secured: but sooner or later the attacks are sure to recur, and when this has happened there is but one certain cure, namely, partial excision of the Gasserian ganglion. As to the method of performing it, Mr. Hutchinson adopts the Hartley-Krause plan of approach, with the patient in the sitting posture to diminish bleeding; and he removes only the inferior portion of the ganglion with the lower two nerve roots. His reasons for this are that the operation is easier, the ophthalmic division of the fifth is seldom involved, and may therefore be preserved, while its destruction is frequently followed by destructive lesions in the eye to which it is distributed. The dangers and difficulties of the various extracranial and intracranial operations which have from time to time been suggested, are well described, and we can confidently commend the book to the notice of every practical surgeon.

AUTO-BIOGRAPHY OF FREDERICK JAMES GANT, F.R.C.S., Consulting Surgeon to the Royal Free Hospital. (London: Baillière, Tindall and Cox. 1905. Pp. 200. Price 3s. 6d. net.)

MR. GANT in this quaintly expressed, curiously constituted volume records the doings and impression of a life devoted to human interests, the furtherance of moral purposes, and the progress of medical science. The author writes with the mellow egotism of an octogenarian. There are here reflected matters concerned with authorship, hospital experiences, and public duties. Certain pages, however, manifest a spirit of animosity which the friends of the author cannot but regret, and there are references to personal and private affairs which are calculated to give pain. The work will be read with interest by Mr. Gant's old students and many friends, but the volume is hardly likely to reconcile the majority of readers to the auto-biographical method of personal history.

THE HOLY COMMUNION: A POSSIBLE SOURCE OF INFECTION. By HENRY BLANDY, L.D.S. Ed. (Nottingham: J. and H. Bell, Ltd. 1905. Pp. 12. Price 6d.)

THIS well-intentioned but loosely constructed brochure seeks to awaken attention to the possibility of communicating such diseases as tuberculosis, diphtheria, syphilis, and infectious catarrhs by means of the communion cup. The chief interest, however, centres in certain letters which the author has received from well-known leaders of the ecclesiastical and medical worlds approving his efforts to procure a more hygienic conduct of the practice of communion. Mr. Blandy has done well in focussing attention on a matter needing a wisely directed reform.

THE CRUSADE AGAINST PHTHISIS. By A. B. DE GUERVILLE. (London: Hugh Rees, Limited. 1904. Price 6d.)

THIS brochure is a tribute from a grateful patient to Dr. Walther and his sanatorium at Nordrach, and it is chiefly occupied with a record of the author's personal experiences at various health resorts. There is nothing in the booklet of special interest to medical men, but it will doubtless be of service in educating the public to appreciate the benefits to be derived from the sanatorium treatment of consumption.

THE PRESENT CONDITIONS OF INFANT LIFE, AND THEIR EFFECT ON THE NATION. Published for The Infants' Health Society. (London: Baillière, Tindall and Cox. 1905. Price 6d.)

THE object of this pamphlet is to draw public attention to the deleterious effect upon the community of improper methods of infant feeding. No well-informed person will deny the magnitude or the pressing nature of the evil; and like every other attempt to educate public opinion towards reform this convincing essay is welcome.

---

### BOOKS RECEIVED.

BAILLIÈRE, TINDALL AND COX.

